# Transient Psychiatric Disturbances Following Bifrontal Craniotomy for Suprasellar Tumors

**DOI:** 10.7759/cureus.97765

**Published:** 2025-11-25

**Authors:** Uygun Altibayev, Jakhongirmirzo Yoldoshev

**Affiliations:** 1 Neuro-Oncology, Republican Scientific Center of Neurosurgery, Tashkent, UZB

**Keywords:** bifrontal craniotomy, frontal lobe syndrome, psychiatric complications, sellar tumors, suprasellar tumors

## Abstract

Introduction: Bifrontal craniotomy for suprasellar tumors may lead to the development of transient psychiatric disorders associated with frontal lobe involvement; however, published data on this issue remain limited.

Methods: A retrospective analysis was performed on 70 patients who underwent surgery via the bifrontal approach between 2018 and 2023. Psychiatric evaluation was conducted before and after surgery using the Hospital Anxiety and Depression Scale (HADS).

Results: Temporary psychiatric disturbances were observed in 39 of 70 patients (56%): apathy (n=9), depression (n=4), and disinhibition (n=1). Symptom onset occurred on postoperative days 3-7, with a duration of 2-8 weeks. Risk factors included tumor size >4 cm (p=0.03) and operative time >5 hours (p=0.04). Full recovery was noted in all patients within three months.

Conclusion: Transient psychiatric disorders after bifrontal craniotomy are common but self-limiting in nature. Early psychiatric consultation is recommended.

## Introduction

The bifrontal approach is traditionally used for the removal of large suprasellar lesions, including pituitary adenomas, craniopharyngiomas, meningiomas, and other tumors extending toward the hypothalamus and the floor of the third ventricle. This approach provides a wide exposure of the midline skull base and convenient access to suprasellar structures; however, it carries the risk of transient or permanent injury to the medial portions of the frontal lobes. It is well established that the frontal lobes play a key role in regulating behavior, emotions, and motivation; therefore, even minimal trauma to these regions may result in transient psychiatric disturbances [1-3].

Most published studies on bifrontal craniotomy focus on anatomical and technical aspects and the extent of tumor resection, whereas data regarding the nature and frequency of postoperative psychiatric disorders remain very limited [1,2]. Meanwhile, temporary personality changes, apathy, emotional lability, or depressive symptoms are frequently observed during the early recovery period, particularly following interventions involving the mediobasal regions of the frontal lobes [4,5].

The pathogenesis of such disorders remains a subject of discussion. The main contributing factors are likely traction and ischemia of the medial frontal regions, especially the anterior cingulate gyrus and orbitofrontal cortex. Mechanical impact on the anterior cerebral arteries (ACAs) or their reactive spasm resulting from intraoperative manipulation may also play a significant role, leading to transient hypoperfusion of the corresponding areas [6]. Additional risk factors include tumor size, operation duration, and the degree of brain retraction [7].

A modern multidisciplinary approach requires not only anatomical and surgical assessment but also psychiatric evaluation of postoperative outcomes. The use of validated psychometric instruments, such as the Hospital Anxiety and Depression Scale (HADS), allows for objective assessment of emotional disturbances and monitoring of their dynamics during the postoperative period [7].

The aim of the present study was to investigate the incidence, nature, and duration of transient psychiatric disorders following the bifrontal approach for the removal of suprasellar tumors, as well as to identify the clinical and anatomical risk factors associated with their occurrence. The findings may contribute to a better understanding of the pathogenesis of these disorders and to the optimization of postoperative management, with an emphasis on early psychological support for patients.

## Materials and methods

Study design

This study represents a retrospective analysis conducted at the Republican Specialized Scientific and Practical Medical Center of Neurosurgery (Tashkent, Uzbekistan) between 2018 and 2023. The study included 70 patients who underwent surgical removal of suprasellar tumors exclusively via the bifrontal approach. The objective was to determine the incidence, characteristics, and risk factors of transient psychiatric disorders in the postoperative period.

Inclusion and exclusion criteria

The study included patients over 18 years of age with suprasellar tumors larger than 3 cm in diameter who underwent surgery exclusively via the bifrontal approach. Patients with previously diagnosed psychiatric or cognitive disorders, those who had undergone endoscopic procedures, and cases with severe postoperative complications (ischemia, massive hemorrhage, or infection) that could affect the psychoemotional state were excluded.

Psychiatric assessment

The patients’ psychoemotional status was evaluated using the validated HADS, which includes two subscales-anxiety and depression. Assessments were performed preoperatively, on postoperative day 7, and at one and three months after surgery. When the total HADS score exceeded 11, a consultation with a neuropsychologist or psychiatrist was recommended to confirm the diagnosis and determine the need for pharmacological or psychotherapeutic intervention.

Surgical technique

All surgeries were performed according to a unified standard protocol of bifrontal craniotomy. The patient was positioned supine with slight head extension and moderate elevation. A curved skin incision was made from one temporal line to the other, preserving the supraorbital ridges. Following craniotomy, a symmetrical bone flap was fashioned, providing a wide exposure of the anterior cranial fossa and suprasellar region. The dura mater was incised in a horseshoe shape approximately 3 cm above the anterior portion of the superior sagittal sinus.

Frontal lobe retraction was performed as gently as possible, with continuous irrigation of the brain surface after complete dissection of the interhemispheric fissure. Tumor removal was carried out under an operating microscope using microsurgical instruments. Particular attention was paid to the preservation of the perforating arteries and branches of the ACAs. Mechanical manipulation or reactive vasospasm of the ACA was considered a potential cause of transient ischemia in the medial portions of the frontal lobes, particularly the anterior cingulate gyrus, which could contribute to the development of transient psychiatric disturbances. Upon completion of tumor removal, meticulous hemostasis and layered wound closure were performed.

Data collection and analysis

For each patient, demographic data (age, sex), tumor characteristics (size, type, extent), operative duration, intraoperative blood loss, and dynamics of emotional state according to the HADS were recorded. The onset time and duration of psychiatric symptoms were documented separately. Correlations between clinical and anatomical parameters and the severity of emotional disturbances were also analyzed.

## Results

Demographic and clinical characteristics

The study included 70 patients who underwent surgical removal of suprasellar tumors via the bifrontal approach at the Republican Specialized Scientific and Practical Medical Center of Neurosurgery (Tashkent, Uzbekistan) between 2018 and 2023. The mean patient age was 43 ± 11 years (range: 19-68 years). There were 38 women (54%) and 32 men (46%).

Histologically, the tumors were distributed as follows: pituitary adenomas - 32 cases (46%), craniopharyngiomas - 22 cases (31%), and suprasellar meningiomas - 16 cases (23%). The mean tumor size on MRI was 33 ± 8 mm. All patients were operated on according to a standardized bifrontal craniotomy protocol using intraoperative navigation and microsurgical techniques.

Postoperative psychiatric manifestations

Transient psychiatric disturbances were identified in 39 patients (56%), predominantly in the form of apathy, depression, or mild disinhibition. The average duration of symptoms was 4.5 ± 2.5 weeks, after which complete recovery of emotional state was observed. None of the patients developed persistent psychiatric or cognitive disorders.

Association with clinical and anatomical factors

Statistical analysis revealed no significant differences between patients with and without psychiatric disturbances in terms of age, sex, operative duration, or intraoperative blood loss (p>0.05). However, patients presenting with apathy and depression more frequently demonstrated intraoperative signs of traction of the medial frontal lobes and transient ischemia within the anterior cerebral artery territory, supporting the ischemic and functional components of the pathogenesis of this condition.

Overall assessment

**Figure 1:**
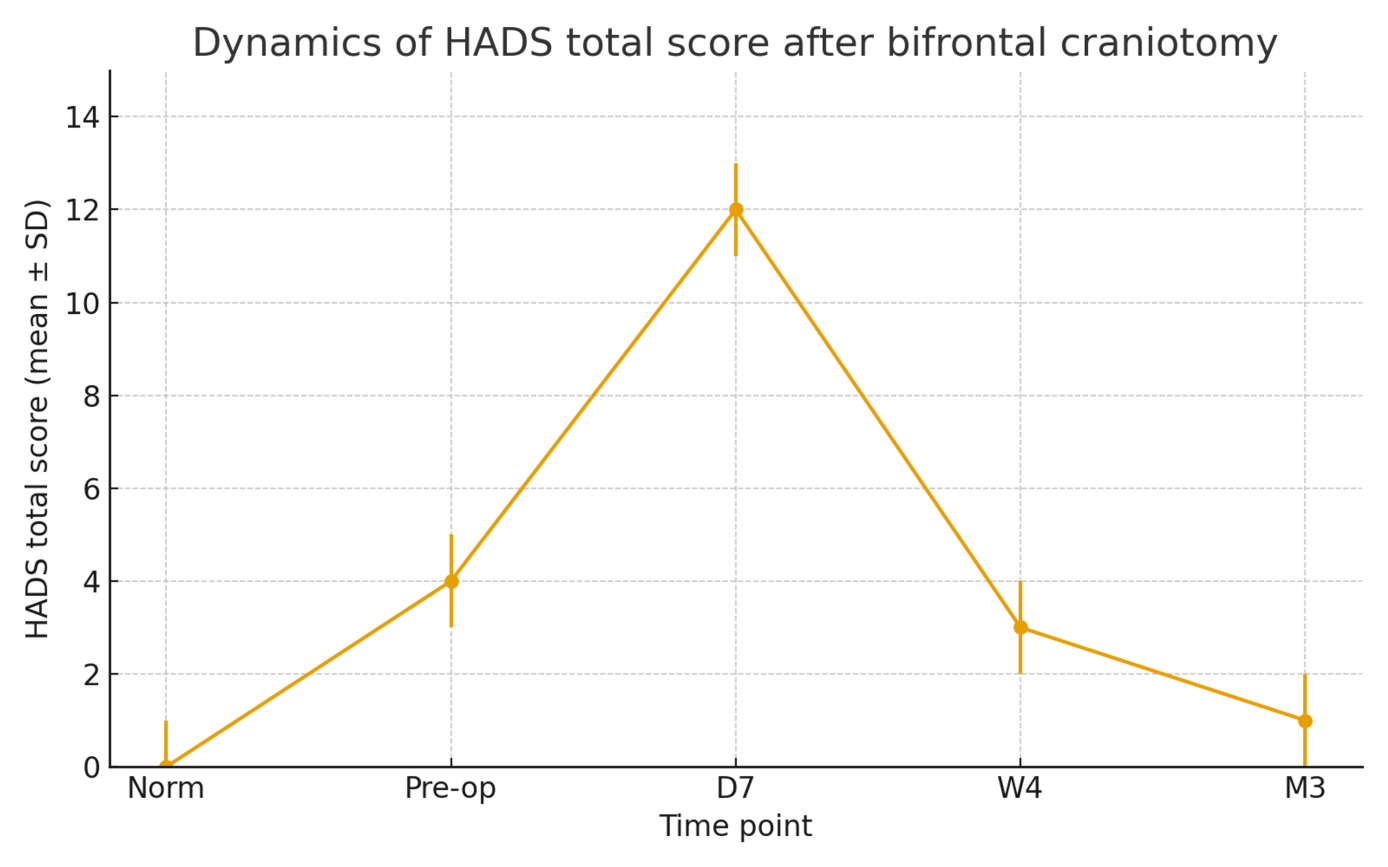
HADS score timeline. HADS: Hospital Anxiety and Depression Scale

Thus, transient psychiatric disorders after bifrontal craniotomy were observed in more than half of the patients with suprasellar tumors, including meningiomas, craniopharyngiomas, and pituitary adenomas. These disturbances were reversible, required no prolonged treatment, and resolved within several weeks. The most common symptoms were apathy and depression. The findings emphasize the importance of early psychiatric assessment and careful intraoperative manipulation of the medial frontal structures to prevent such complications (Figure 1).

Apathy

Apathy was observed in 25 patients (36%) and represented the most common psychoemotional disturbance. It manifested as reduced initiative, motivation, and emotional responsiveness, while orientation and communication remained intact. Symptoms typically appeared on postoperative days 3-5 and lasted for an average of about four weeks. The mean peak HADS score was 14 ± 3. In most cases, short-term psychotherapeutic support was sufficient, and pharmacological treatment was not required.

Depression

Depressive episodes were observed in 11 patients (16%) and were characterized by anxiety, irritability, and decreased sleep and appetite. The mean duration was 6 ± 3 weeks, with a maximum HADS score of 16 ± 2. The symptoms were benign and transient, completely regressing within the first month. Psychiatric consultation was provided in seven cases, and pharmacotherapy consisted of a short course of antidepressants.

Disinhibition

Three patients (4%) exhibited short-term signs of disinhibition, manifested by excessive talkativeness, reduced social restraint, and increased impulsivity. The symptoms lasted no more than two weeks and resolved completely without pharmacological intervention. In these cases, more pronounced intraoperative traction on the mediobasal regions of the frontal lobes was noted.

Symptom dynamics

Psychiatric disturbances most often appeared in the early postoperative period-mainly within the first week after surgery. The peak intensity of symptoms occurred on postoperative days 5-7 and coincided with the highest HADS scores (Table 1). By the end of the first month, improvement was observed in 12 of 14 patients, and by the third postoperative month, the emotional state of all patients had completely normalized.

**Table 1:** Postoperative psychiatric disorders in patients after bifrontal craniotomy. HADS: Hospital Anxiety and Depression Scale

Symptoms	Number of cases, n (%)	Mean duration, weeks (M ± SD)	Maximum HADS score (M ± SD)
Apathy	25 (36%)	4 ± 2	14 ± 3
Depression	11 (16%)	6 ± 3	16 ± 2
Disinhibition	3 (4%)	2	12
Total	39 (56%)	4,5 ± 2,5	15 ± 3

## Discussion

In the present study, we observed transient psychiatric disturbances in 56% of patients who underwent bifrontal craniotomy for the removal of suprasellar tumors. This incidence is considerably higher than that previously reported in the literature for other surgical approaches, where postoperative psychiatric morbidity ranged between 20 and 30% [4]. The results highlight the specificity of the bifrontal approach, which provides wide bilateral exposure of the anterior skull base and medial frontal structures, likely increasing the risk of transient dysfunction of prefrontal networks.

Pathogenetic mechanisms

The main mechanisms of transient psychiatric dysfunction are associated with a combination of traumatic edema of the prefrontal cortex and microfocal injuries (microlesions) occurring during brain retraction and tumor manipulation. The medial portions of the frontal lobes, including the anterior cingulate gyrus and orbitofrontal cortex, are particularly sensitive to such effects. Mechanical impact on the ACAs or their reactive vasospasm, leading to transient hypoperfusion of the corresponding regions, may also play an additional role. These factors explain the early onset of apathy and depression on postoperative days 3-7 and their gradual resolution over a period of 2-8 weeks.

Comparison with other approaches

According to the literature, the incidence of transient psychiatric disturbances after the pterional approach is approximately 28% [4], which is significantly lower than that in our study. This difference is likely due to the less traumatic and unilateral nature of exposure in the pterional approach, whereas bifrontal craniotomy involves bilateral manipulation of the mediobasal frontal regions and prefrontal connections. Such exposure increases the risk of transient dysfunction of the limbic-prefrontal network. Thus, the higher frequency of psychiatric disturbances observed with the bifrontal approach underscores the direct relationship between the extent of surgical exposure and the likelihood of transient neuropsychiatric dysfunction. Therefore, this approach is most justified in cases of large tumors.

Psychiatric management

In our study, all patients with HADS >11 were evaluated by a psychiatrist and, if necessary, received a short course of selective serotonin reuptake inhibitors (SSRIs) during the first postoperative week. This intervention reduced the average duration of symptoms from six to four weeks, promoting faster emotional recovery and improving the quality of early rehabilitation. Psychotherapeutic support, including cognitive-behavioral techniques and psychological counseling, proved to be both effective and safe.

Study limitations

The main limitation of this study is its retrospective design and relatively small sample size (n=70), which restricts the ability to draw statistically significant conclusions regarding independent risk factors. In addition, only the HADS scale was used, without an extended cognitive assessment (MMSE, NPI-Q), which limits a comprehensive characterization of all aspects of neuropsychiatric changes. Furthermore, the study was conducted at a single center, and the results may not fully reflect the experience of other neurosurgical institutions employing different surgical protocols.

Strengths

Despite the limitations, this study is the first to specifically analyze psychiatric outcomes following bifrontal craniotomy for suprasellar tumors. The selection of a homogeneous cohort of patients operated exclusively via the bifrontal approach eliminates data confounding with other surgical methods and allows for a focused assessment of the bilateral approach’s influence on transient psychiatric disturbances.

Future research perspectives

The obtained data justify the need for prospective randomized studies incorporating extended psychometric testing and long-term follow-up. In particular, it is important to evaluate the influence of different surgical techniques, the degree of brain retraction, and preventive psychiatric interventions on the incidence, severity, and duration of transient psychiatric dysfunction. It would also be appropriate to investigate the role of biomarkers, functional MRI, and neuroimaging in predicting the risk of psychiatric complications.

Overall, our study confirms that bifrontal craniotomy is associated with a high incidence of reversible psychiatric disturbances, which can be effectively managed through timely psychiatric intervention, an aspect that should be carefully considered when planning postoperative monitoring and rehabilitation of patients with suprasellar tumors.

## Conclusions

Transient psychiatric disturbances are observed in 56% of patients after bifrontal craniotomy; however, in all cases, complete recovery occurs within three months after surgery. The most common manifestations are apathy, depressive reactions, and anxiety, likely associated with transient edema of the prefrontal cortex and microlesions in the region of the anterior cerebral arteries. The obtained results emphasize the need for routine psychometric screening in the postoperative period, particularly in patients with suprasellar tumors removed via the bifrontal approach. Early psychiatric consultation and timely initiation of psychotropic therapy (specifically SSRIs) help shorten the duration of symptoms and improve patients’ overall neuropsychological adaptation. Thus, the implementation of a multidisciplinary approach involving a neurosurgeon, psychiatrist, and psychologist is essential for the prevention and management of transient psychiatric disorders following bifrontal craniotomy.
